# A Foraging Cost of Migration for a Partially Migratory Cyprinid Fish

**DOI:** 10.1371/journal.pone.0061223

**Published:** 2013-05-28

**Authors:** Ben B. Chapman, Anders Eriksen, Henrik Baktoft, Jakob Brodersen, P. Anders Nilsson, Kaj Hulthen, Christer Brönmark, Lars-Anders Hansson, Peter Grønkjær, Christian Skov

**Affiliations:** 1 Department of Biology, Aquatic Ecology Unit, Lund University, Lund, Sweden; 2 National Institute of Aquatic Resources, Technical University of Denmark (DTU), Silkeborg, Denmark; 3 Department of Fish Ecology and Evolution, EAWAG Swiss Federal Institute of Aquatic Science and Technology, Centre of Ecology, Evolution and Biochemistry, Kastanienbaum, Switzerland; 4 Department of Bioscience, Aarhus University, Aarhus, Denmark; University of Western Ontario, Canada

## Abstract

Migration has evolved as a strategy to maximise individual fitness in response to seasonally changing ecological and environmental conditions. However, migration can also incur costs, and quantifying these costs can provide important clues to the ultimate ecological forces that underpin migratory behaviour. A key emerging model to explain migration in many systems posits that migration is driven by seasonal changes to a predation/growth potential (p/g) trade-off that a wide range of animals face. In this study we assess a key assumption of this model for a common cyprinid partial migrant, the roach *Rutilus rutilus*, which migrates from shallow lakes to streams during winter. By sampling fish from stream and lake habitats in the autumn and spring and measuring their stomach fullness and diet composition, we tested if migrating roach pay a cost of reduced foraging when migrating. Resident fish had fuller stomachs containing more high quality prey items than migrant fish. Hence, we document a feeding cost to migration in roach, which adds additional support for the validity of the p/g model of migration in freshwater systems.

## Introduction

Animal migration is a spectacular and ecologically important phenomenon. It is also taxonomically widespread, with animals from all major vertebrate groups (fish [1], amphibians [2], reptiles [3], mammals [4], birds [5]) and many invertebrates (odonata [6], lepidoptera [7], crustacea [8], mollusca [9]) adopting migration as a strategy to maximise fitness in the face of predictable temporal changes to habitat quality. A variety of ecological/environmental forces have been implicated in driving the evolution of migration, and animals are thought to migrate for a number of reasons, for example to escape seasonally adverse weather conditions or avoid predators [10], [11]. Whilst there are clear benefits to migration, there can also be costs. Migratory journeys can be energetically arduous, and costly in terms of the distance travelled, especially for long-distance migrants [10]. For example, migratory Atlantic salmon *Salmo salar* can expend up to 60–70% of their energy reserves during their spawning migration [12], and both sustained flight and also stop-overs are costly for migratory songbirds, especially in cool weather [13]. Costs of migration can also take the form of increased risk of mortality due to a heightened predation risk along the route [14], exposure to novel parasites and pathogens that occur at the migratory destination [15], or in terms of reduced food availability/quality [16]. Quantifying the costs of migration is important to gain insights into the evolutionary processes which underlie migratory behaviour, and also to test ecological trade-off models of migration [17].

It is increasingly apparent that migration as a strategy can be a product of trade-offs which fluctuate in a predicable way over time [16]. These insights are often produced by studies into partially migratory populations, i.e. populations that consist of both migratory and resident individuals [17]. Partial migration provides an excellent opportunity to quantify the costs and benefits of migration, and hence to test ecological trade-off models of migration [17], [18]. One prevalent trade-off thought to govern migratory dynamics across a range of species is the predation risk/growth potential (p/g) trade-off. In this p/g model seasonal (or daily) shifts in the strength of the trade-off drive migratory habitat shifts [16]. Data suggests that this model may explain diverse migratory phenomena including diel vertical migrations in zooplankton and fish and seasonal migrations in ungulates and cyprinid fishes [16], [17], [19], [20], [21]. The p/g model as applied to partial migrants such as cyprinid fishes, that migrate from lakes to streams during winter, predicts that residents pay a cost in terms of a high predation risk in the lake but benefit via higher food availability and hence growth potential, whilst migrants benefit from reduced predation risk in the stream but pay a cost migrating to a food-poor habitat [17], [18]. However, data to test these axioms is lacking. For example, whereas theoretical predictions of migratory patterns in cyprinid fish derived from the p/g model are closely matched by data on the cyprinids' seasonal movements [16], empirical evidence of differences in food availability/quality between habitats is absent. In this study, we present data to assess a foraging cost to migration in a common cyprinid fish, roach, *Rutilus rutilus* ¸ a freshwater partial migrant.

Seasonal migration in roach is widespread across Europe [22], [23], [24]. Individuals migrate from shallow lakes during autumn and overwinter in connected streams before returning to the lake in spring [25]. Despite a great deal of indirect evidential support for the p/g model for roach migration [16], [21], [26], and also direct evidence of differences in predation risk for migrants and residents in this system [27], no study has shown that there is a foraging cost for migrants overwintering in stream habitats compared to residents that remain in the lake. Hence a key assumption of the p/g model remains untested in this system. Here we present data to test if there is a foraging cost to winter migration in roach, via lower gut fullness and also in terms of diet quality. We sample migrants and residents during the migratory period and compare both the gut fullness and diet composition of migrants and residents to test the hypothesis that there is a feeding cost to migration in roach.

## Materials and Methods

### Sampling

**Figure 1:**
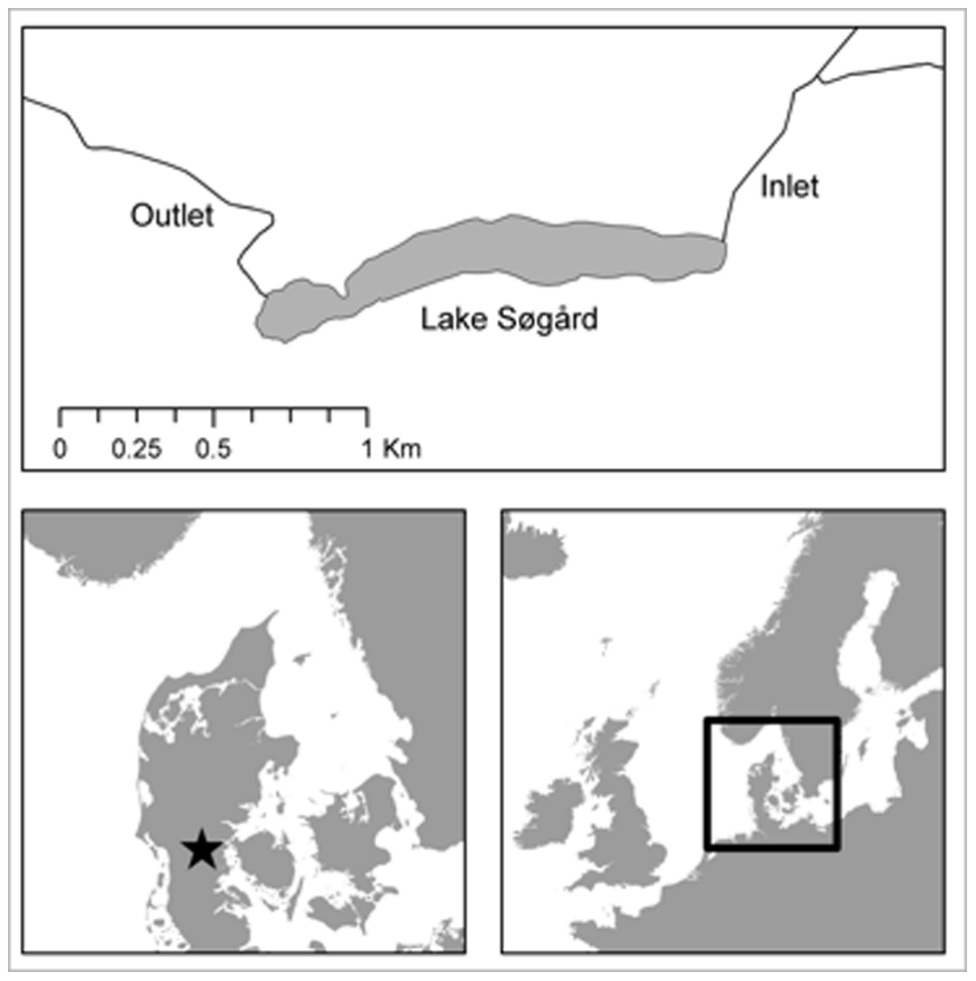
Map showing the location of Lake Søgård.

Roach were sampled from Lake Søgård in Denmark, and its inlet tributary during the migratory period, i.e. mid-October 2010 to late March 2011. Lake Søgård (55°29' N, 9°19' E) is a small, eutrophic and shallow lake (area 26 ha; average depth 1.6 m; mean summer Secchi depth 0.55 m) with a well-defined inlet and outlet (Fig. 1). The fish community is dominated by roach and Eurasian perch (*Perca fluviatilis*). Additionally, common bream (*Abramis brama*), rudd (*Scardinus erythrophthalmus*), white bream (*Blicca bjoerkna*), pike (*Esox lucius*) and European eel (*Anguilla anguilla*) occur in this lake. Submerged vegetation is largely absent and a 3–4 m wide margin of emergent vegetation dominated by common reed (*Phragmites australis*) borders the lake.

Each habitat (lake and inlet stream) was sampled four times using a combination of electrofishing and gill nets. Sampling took place in autumn on the 19^th^ October (lake and stream), 2^nd^ November (lake and stream), 11^th^ November (lake and stream), and in spring, on the 24^th^ March (lake) and 28^th^ March (stream). Immediately following capture, fish were weighed to the nearest 0.1 g and measured to the nearest 0.1 cm (n = 340). A subsample were euthanized with an overdose of benzocaine and, while kept on ice, transported to the laboratory for gut content analysis (n = 132).

### Gut content analysis

Individual gut fullness was visually assessed and categorised as 0 %, 1–5 %, 6–25 %, 26–50 %, 51–75 % or 76–100 % (Table 1). Gut contents were subsequently rinsed through a 90 µm filter. The remaining material was examined under a 50X stereo microscope and classified for analysis as zooplankton, molluscs, detritus, plant material or invertebrates.

**Table 1:** Gut fullness data.

Gut fullness score (% full)	Autumn		Spring	
	Residents	Migrants	Residents	Migrants
0 (0%)	2	7	4	14
1 (1–5%)	5	7	4	3
2 (6–25%)	6	4	10	3
3 (26–50%)	7	9	0	0
4 (51–75%)	6	6	2	0
5 (76–100%)	20	13	0	0
TOTAL	46	46	20	20

We further classified food types as being low or high quality. Animal food (i.e. zooplankton, molluscs and invertebrates) were classed as high quality food resources, and detritus and plant material as low quality food resources, as assimilation efficiency is much lower for plant compared to animal food in roach [28].

### Body condition

Length and weight were measured from the assayed fish in order to calculate body condition. We used Fulton's condition factor, which is a commonly used index of fish condition, calculated as *F* = (100*M*) *L* ^–3^, where *M* is mass in grams and *L* is total length in centimetres. As *F* increases with body size in roach [26] we used the residuals from the regression between *F* and *L* as an estimate of length-specific condition.

### Population patterns of migration

We also carried out a study to describe patterns of population migration into the streams. We monitored migration by passive telemetry using a modified PIT-tag antenna system [24], [29]. Firstly, in September 2010 prior to migration, fish were captured via electrofishing and individuals tagged following [29] by surgically implanting a TIRIS passive integrated transponder tag (PIT tag) (Texas Instruments, RI-TRP-RRHP, Plano, Texas, USA; half duplex, 134 kHz, 23.1 mm long, 3.85 mm diameter, 0.6 g in air) into the stomach cavity of the fish (N = 299; total length: 125–250 mm). Tagged individuals were then released back into the lake and their migratory movements monitored using passive telemetry. Previous work has shown that there are no significant effects of tagging upon fish well-being, such as body condition [29]. Antennae were installed in the streams connected to the lake. When a tagged fish swims past an antenna, the PIT-tag emits a unique code that is recorded and stored along with the date and time of passage. Two loop-shaped antennae were placed in all connected streams, which allowed us to determine fish swimming direction. The recording frequency was set to 5 energise/receive cycles per second, and migration data were collected from the time of tagging until June 1^st^ 2011.

### Statistics

#### Gut fullness

To analyse gut fullness differences between migrants and residents we assigned each fish a score (0–5) based on the degree of gut fullness categories above (Table 1). We then contrasted gut fullness for lake residents and stream migrants using a non-parametric Mann-Whitney test. We carried out additional analysis of gut fullness to test for differences in autumn and spring separately, to assess the effect of migration in different time periods. All fish caught between 19^th^ October –11^th^ November were categorised as autumn samples, and fish caught between 24^th^ –28^th^ March were designated as spring samples.

#### Food type variation in diet

To test for differences in food types in the diets of migrant and resident fish we first contrasted all migrant versus all resident fish, and then separately analysed autumn and spring fish. To analyse the data we used a Chi squared test against a null model that food item presence in guts should be equivalent between habitats if there are no differences in food types eaten between the lake and the stream. In other words we calculated expected values for migrants and residents by dividing the total number of fish found with a given food item in their stomach by two. We analysed all gut contents, and also separately analysed ‘high quality’ food item presence.

Finally, we compared condition between migrant and resident fish, and autumn and spring caught fish using Mann-Whitney tests, as this data did not conform to assumptions of normality required for parametric analysis. We analysed using both data from our sampling to compare the condition of migrants and residents in autumn and spring, and also using our telemetry study to compare the condition of migrants and residents prior to the onset of migration (i.e. when fish were tagged in September).

We also report effect sizes for all instances where P<0.1 [30], which were calculated using the formula
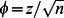
for Mann-Whitney analyses, where *z* is a test statistic and *n* denotes sample size and



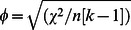
for Chi squared tests, where *n* is the number of samples and *k* is the the lower value of either the number of rows or columns.

Mann-Whitney analyses were carried out in SPSS, and Chi squared test values were calculated in Excel.

#### Ethics statement

All field sampling was carried out with the relevant permissions from the Ministry of Food, Agriculture and Fisheries of Denmark. Tagging was carried out with permission from the Danish Experimental Animal Committee.The study did not involve endangered or protected species.

## Results

### Gut fullness

**Figure 2:**
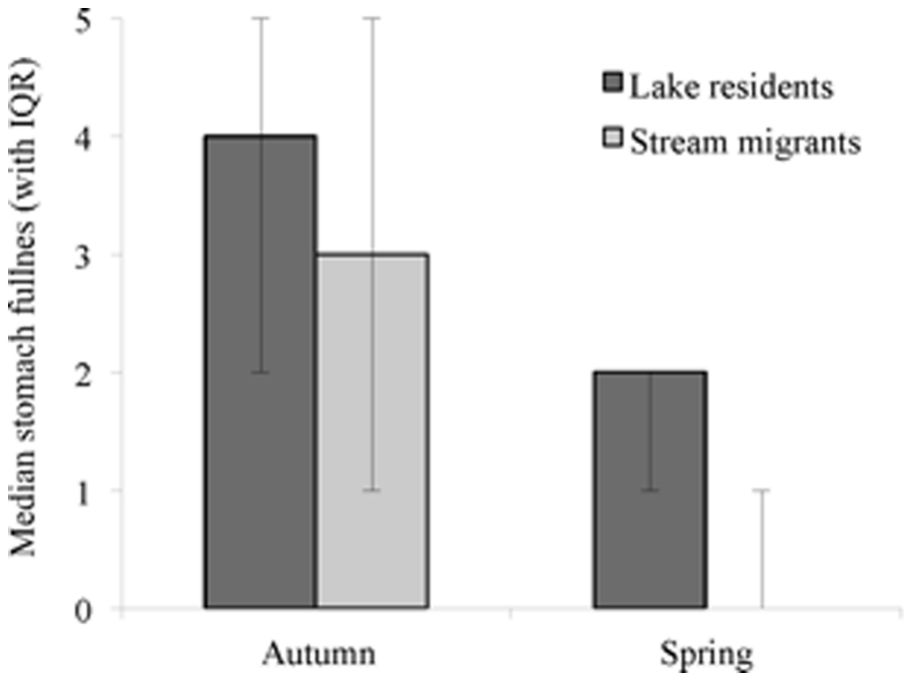
Median gut fullness of lake resident and stream migrant fish in autumn and spring. Error bars indicate interquartile range values.

Fish had fuller guts in autumn compared with spring (Mann-Whitney test: U = 630, z = −6.1, N = 132, P<0.001, φ = 0.53). This pattern was also evident irrespective of migratory type (Mann-Whitney test: residents: U = 168, z = −4.176, N = 66, P<0.001, φ = 0.51; migrants: U = 128.5, z = −4.739, N = 66, P<0.001, φ = 0.58). The degree of gut fullness differed between migrants and residents (Mann-Whitney test: U = 2796, z = −2.588, N = 132, P = 0.01, φ = 0.22), with residents having significantly fuller guts than migrants. In spring residents (i.e. lake occupants) had significantly fuller guts than stream-occupying migrants (Mann Whitney test: U = 85, N = 40, P = 0.001, φ = 0.53: Fig. 2) with similar but marginally non-significant differences already evident in autumn (Mann-Whitney test: U = 833, z = −1.81, N = 92, P = 0.07, φ = 0.18: Fig. 2).

### Food type variation

**Figure 3:**
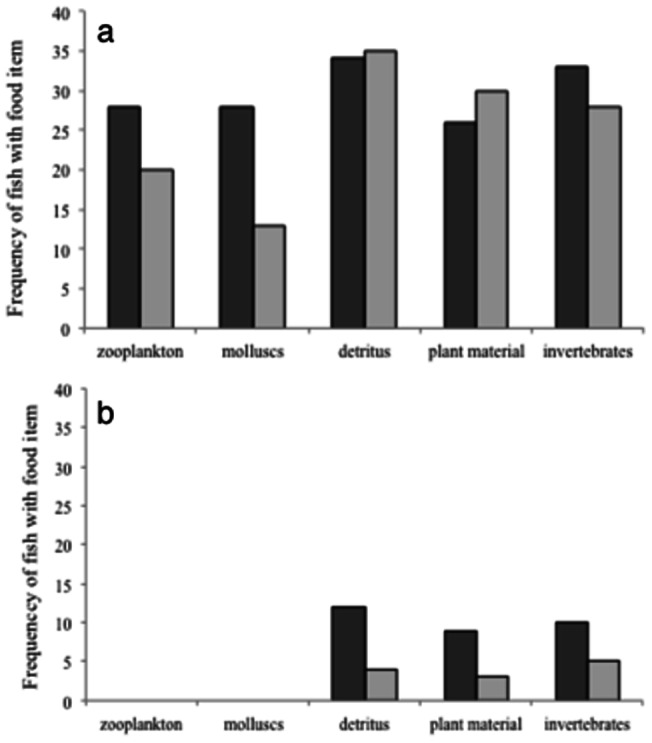
Dietary differences between lake resident (dark grey bars) and stream migrant (light grey bars) roach. The bars indicate the frequency that different food types were found within the guts of sampled fish from the different habitats in (a) autumn and (b) spring.

The frequency of individuals found with the different prey items in their guts also varied between migrants and residents, but this was dependent upon food type and season (Fig. 3a & b). In autumn food from all 5 categories was found in roach guts, compared to spring, where zooplankton and molluscs were absent from the guts of all fish. Analysis revealed no significant difference in gut contents in autumn between migrants and residents (χ^2^ = 7.53, *d* = 4, P = 0.11: Fig. 3a) but a significant difference in spring (χ^2^ = 8.67, *df*  = 2, P = 0.01, φ = 0.47: Fig. 3b). Comparing observed and expected values indicated that in spring more migrants than expected by chance had all three food types recorded in this season (detritus, plant material and invertebrates) compared to residents. Including fish from all seasons and all food types in the analysis showed that there overall was a marginally non-significant difference in prey items between migrants and residents (χ^2^  = 8.77, *df*  = 4, P = 0.067, φ = 0.26). Analysing just ‘high quality’ food types (zooplankton, molluscs and invertebrates) for all fish indicated that residents were more frequently found with high quality food items in their guts (χ^2^  = 8.13, *df*  = 2, P = 0.017, φ = 0.25). There were also season-dependent differences between migrants and residents in the number of high quality food items found in their guts. In autumn residents had more high quality food types in their guts than expected by chance (χ^2^  = 7.23, *df*  = 2, P = 0.027, φ = 0.28: Fig. 3a). During spring more residents (n = 10) were found with high quality food (invertebrates) in their guts than migrants (n = 5); however, this difference was not statistically significant (χ^2^  = 1.667, *df*  = 1, P = 0.2).

### Body condition

From our telemetry study, we compared the body condition of eventual migrants and residents prior to the onset of migration in September, and find that migrants were in better condition than residents (Mann Whitney test: U = 5731.5, z = −2.403, P = 0.016, φ* = *0.14). However, our analyses of fish sampled in autumn and spring showed no significant difference in body condition between migrants and residents (Mann-Whitney test: U = 13972, P = 0.81), or fish caught in autumn and spring (Mann-Whitney test: U = 13138, P = 0.43). Further, within-season there was no difference in body condition between migrant and resident fish, either in autumn (Mann-Whitney test: U = 4764, P = 0.27) or spring (Mann-Whitney test: U = 1958, P = 0.4).

### Population patterns of migration

**Figure 4:**
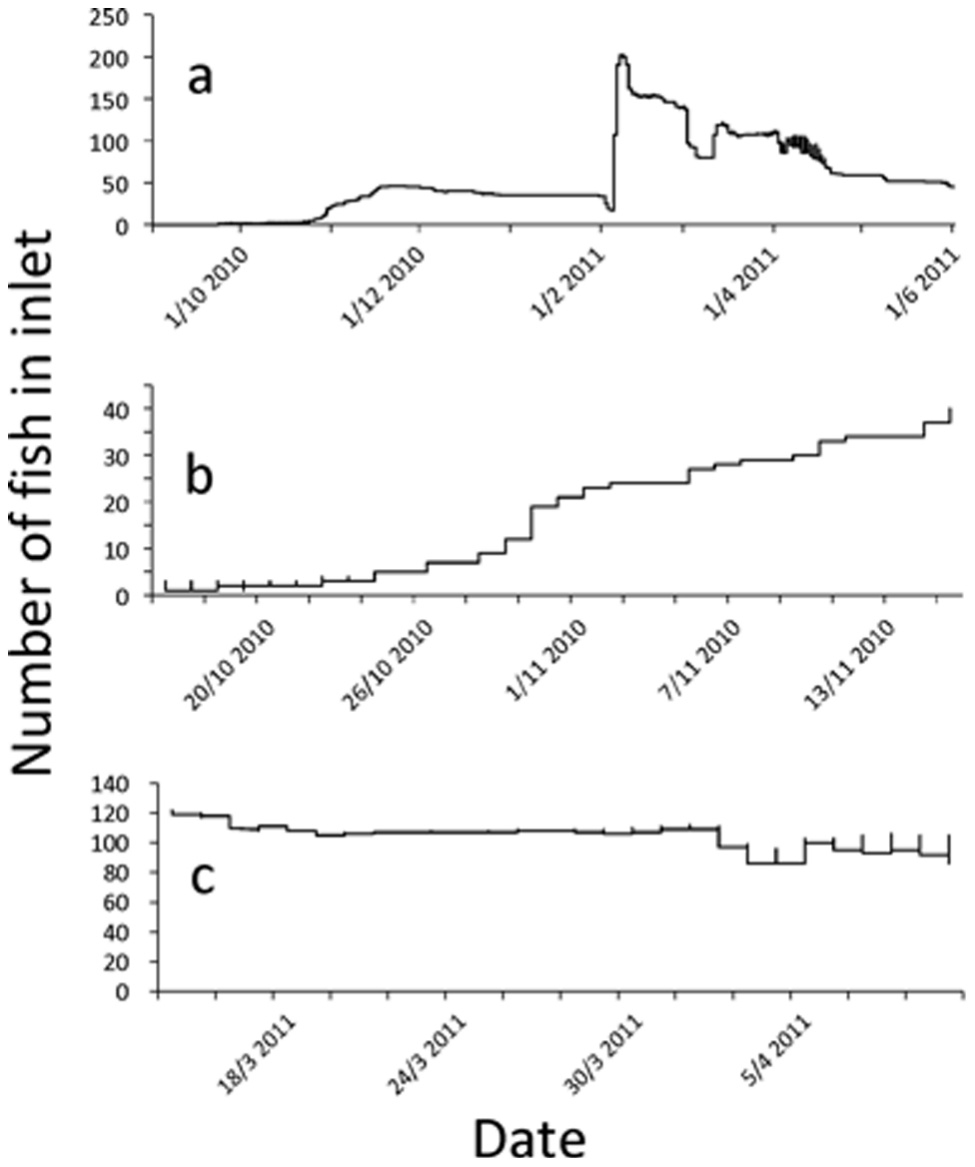
Population patterns of seasonal migration into the inlet stream, for (a) the entire migratory period, (b) the autumn sampling period and (c) the spring sampling period.

We show seasonal patterns of migration into the inlet (Fig. 4a), and also zoom in to highlight patterns of movement around the autumn (Fig. 4b) and spring (Fig. 4c) sampling period. Diel movements between the lake and the stream occur particularly at the beginning and end of the migratory period, but were infrequent throughout our sampling period, and are only present at very low rates on the first sampling occasion (19^th^ October). Especially in the spring sampling period (late March), there is very little movement between habitats, highlighting that we can assign fish to migrant or resident status with high certainty. However, as we lack individual migratory history data for fish that were sampled for gut contents and condition, it is possible that a small number of fish caught in the lake were early return migrants, which means that our analyses are somewhat conservative.

## Discussion

Here we provide the first direct evidence that migratory roach pay a feeding cost, both via reduced feeding (i.e. less full guts), and also in terms of foraging on different food types of differing quality. This supports the predation/growth potential model of cyprinid seasonal migration proposed by Brönmark et al. [16] which builds on previous work on factors determining optimal habitat shifts by Werner and Gilliam [31]. We provide empirical evidence which supports a key assumption of this model as applied to migration: that the growth potential in the low predation habitat is potentially lower due to reduced foraging rates. Hence whilst migration has clear benefits for cyprinids in terms of predator avoidance (against both avian and fish predators: [20], [27], migratory individuals must pay a cost of reduced feeding during winter. This cost may help explain why the migration is only partial, i.e. why only some and not all fish from this and other roach populations migrate during winter. Migration is energetically costly and roach in experimentally induced poor condition (via reduced feeding in the run up to migration) are less likely to migrate than fish in good condition [26]. Recent data from field monitoring corroborates this finding in natural conditions (Brodersen et al. unpublished data). Hence this cost to migration can constrain individuals in poor condition, forcing them to adopt a resident strategy, which contributes to, but does not entirely explain patterns of partial migration in roach [21], [26]. Fish in poor condition may also risk starvation during winter, and hence migrating to a habitat with reduced foraging opportunities can also carry a potential survival cost.

Fish had fuller guts in autumn than in spring, indicating that, for all fish, foraging opportunities declined during winter, and further, gut fullness was lower in stream migrants than lake residents in both seasons. This is strongly suggestive that feeding opportunities are worse in the streams than the lake. Our analyses show particularly strong effects of migratory status upon gut fullness in spring, highlighting the biological significance of this finding. This may be driven by differences in food availability between habitats, or alternatively by increased competition in the streams during winter compared with the lake. We also show that migrants and residents vary in the abundance of different food types in their diet. In autumn, we demonstrate differences between migrants and residents in the number of individuals having recently foraged upon high quality food items such as zooplankton, molluscs and invertebrates, which highlights that, even though the difference in gut fullness is only marginally significant at this time, there is a cost to migrants in terms of a reduced access to a high quality diet. Analysis of the effect size here revealed a moderate effect size (φ = 0.28). It is likely that if access to high quality food is constrained in migrants during autumn this may have consequences for roach, and future work could examine the role of dietary quality in performance, growth and fitness outcomes in cyprinids. In spring, most of the high quality food is entirely absent from roach guts, indicating that all fish have a lower quality diet during winter. However, more residents were found with more of all remaining food types (invertebrates, detritus and plant matter) in their guts compared to migrants, again supporting a foraging cost to migration. Our data also indicates seasonal differences in diet composition, with zooplankton and molluscs being present in roach guts only during autumn. By spring, low quality food played a more dominant role in the diet of lake residents, which shows the seasonal variation in food availability even for residents. With our data we cannot determine whether these differences reflect differences in food availability, or another factor (such as competition). However, irrespective of the precise mechanism, the differences we report in food item presence in the guts of migrants and residents reflect a feeding cost to migration.

We also show differences in initial body condition (i.e. prior to migration) between migrants and residents in our telemetry study. However, we find no differences in body condition between migrants and residents in either autumn or spring. This may indicate a condition cost to migrants, as our analysis of the condition of migrants and residents prior to migration shows that migrants are initially in better condition than residents. As we do not find a difference between migrants and residents during the migratory period this suggests that the condition advantage migrants have initially is soon lost. This finding supports previous research in which individuals experimentally supplemented with food had a better condition, and a higher migratory probability, than those that were fed only limited food that were in worse condition [26], However, it is interesting that at no point in our sampling do residents have better condition than migrants. There is a possibility that there are other costs associated with reduced feeding, for example migrants may have less energy to invest in reproduction, although this is currently speculative. Work to assess the consequences of the foraging cost we report here is ongoing.

Our data adds support for the p/g model in this system. This kind of ecological trade-off is relevant for many taxa in addition to roach and other cyprinids. For example, data on partially migratory ungulates such as elk *Cervus elaphus* has shown that migrants reduce predation risk in return for lower quality forage on the migratory range [19]. Diel vertical migration (DVM) in zooplankton also fits the p/g model, as high quality feeding habitats near the water surface are also more risky [8]. Many fishes exhibit DVM, and size-structured patterns of partial DVM could also be explained by the p/g model [32], although here empirical evidence is required to fully evaluate this possibility. Other migratory fishes also trade-off predation and growth potential: for example partially anadromous brown trout *Salmo trutta* face the same trade-offs, only in reverse. Migrants move to highly productive and risky marine habitats whilst freshwater residents grow more slowly but are thought to face a lower risk of predation [33].

In our system, a clear next step is to compare the growth trajectories of migratory versus resident fish to calculate growth potential directly. This may be possible by combining long-term telemetry monitoring of individuals (to assess migratory history) with otolith analysis to calculate size at age. Until this logistically demanding data is collected, however, our data provides strong support for the validity of a key assumption of the p/g model of seasonal migration in roach, and demonstrates a feeding cost for migration in this species.
